# Deep palmar thermal burns in children: predictive factors of post‑burn contracture and timing of secondary surgery - a cohort study of 273 palms

**DOI:** 10.1016/j.jpra.2026.09.032

**Published:** 2026-10-01

**Authors:** Ugo Lancien, François Thuau, Mathilde Creusat, Estelle Elie, Pierre Perrot

**Affiliations:** a Department of Plastic and Reconstructive Surgery, University Hospital of Nantes, 1 place Alexis Ricordeau, Nantes, 44000, France; b RMeS: Regenerative Medicine and Skeleton research unit - Inserm, Nantes University, 1 place Alexis Ricordeau, Nantes, 44000, France; c Physical rehabilitation department: Centre mutualiste de Kerpape, Ploemeur, 56270, France

**Keywords:** Deep palmar burns, Scar contractures, Pediatric burns, Burn sequelae

## Abstract

**Introduction:**

Deep palmar burns in young children frequently cause disabling contractures. We aimed to identify the incidence, risk factors, and optimal timing of secondary surgery for surgically significant palmar contracture after split-thickness skin grafting in children under five years.

**Materials and methods:**

We conducted a retrospective study of all children <5 years with deep palmar thermal contact burns requiring skin grafting at a single pediatric burn center (2006–2021, minimum 5-year follow-up). All palms were uniformly resurfaced with thin, non-glabrous split-thickness skin grafts harvested from the scalp under a single institutional protocol.

Exclusions included non-contact or dorsal injuries, incomplete data, and loss to follow-up. The primary outcome was scar contracture with functional impairment requiring corrective surgery; time to intervention was secondary. Multivariable Cox regression and Kaplan-Meier analysis identified risk factors.

**Results:**

Across 273 burned palms in 229 children (mean age at burn 16.5 months, 55.7% male), 125 hands (45.8%) developed a palmar contracture requiring secondary surgery. After adjustment, first web space involvement (HRa 4.45) and third-degree depth (HRa 1.93) were independent predictors, and metacarpal head involvement was protective (HRa 0.32). Referral to a specialized rehabilitation center was also associated with contracture (HRa 1.95) but is best interpreted as a marker of initial burn severity rather than an independent predictor. Mean time to surgery among affected cases was 3.2 years, earliest for hypothenar and first web space involvement.

**Conclusion:**

Surgical contracture is common after deep palmar burns in young children, especially with first web space involvement and severe burns. Early risk stratification enables targeted surveillance and intervention, and may help improve patient outcomes. These findings apply to palms resurfaced with thin split-thickness skin grafts; the uniform protocol did not allow comparison with full-thickness or glabrous grafts, which remain alternative options.

## Highlights

• Palmar contractures developed in 45.8% of 273 grafted hands in young children.
• First web space was the strongest predictor (HRa 4.45) of scar contracture.
• Third-degree depth predicted contracture and referral reflected initial severity.
• Metacarpal head involvement reduced risk, suggesting a protective pattern.
• Contractures appeared early with mean surgery at 3.2 years, supporting follow-up.

## Graphical abstract

**Figure:**
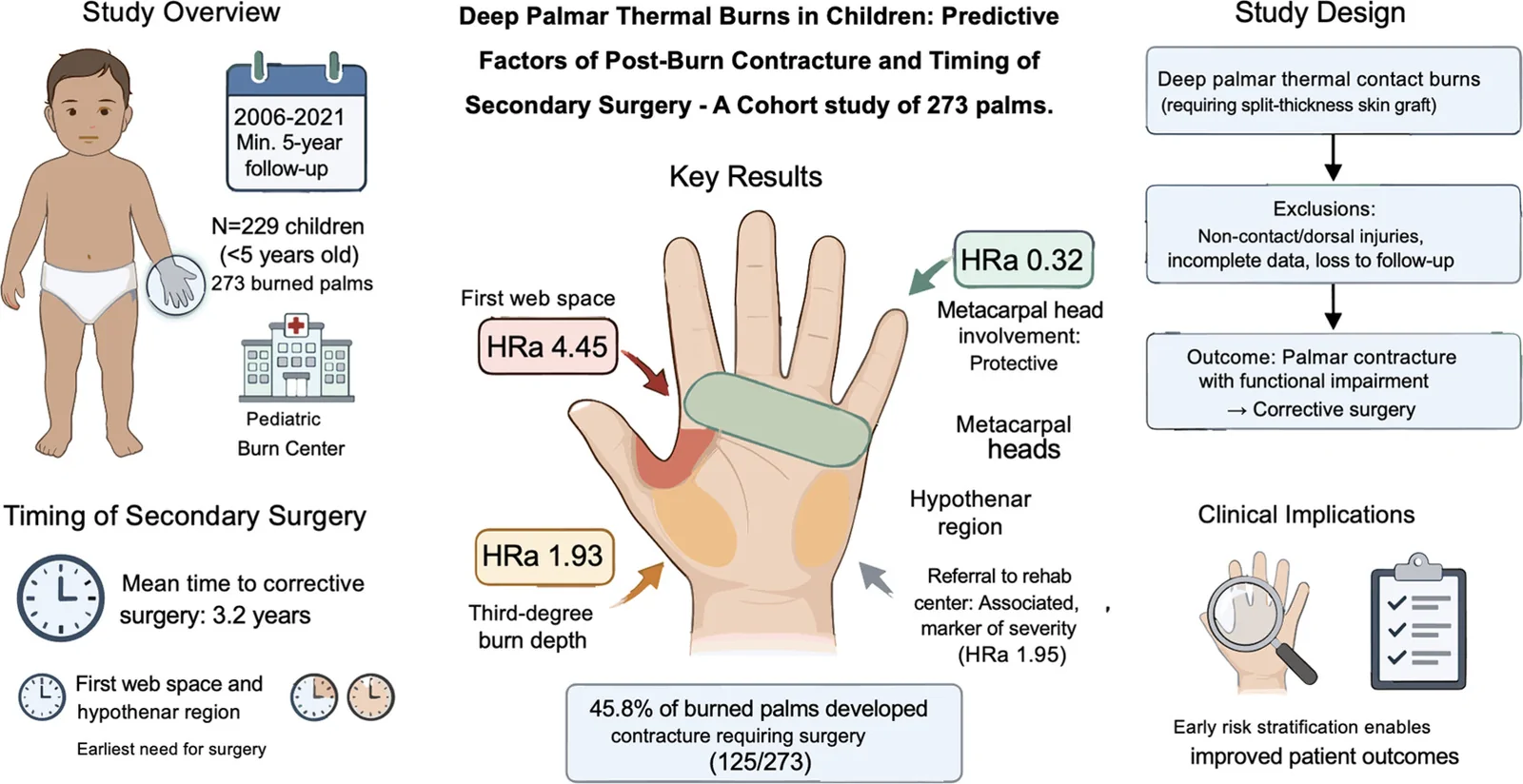

## Introduction

Post-burn hand contractures represent a severe and common complication following deep thermal injuries in the pediatric population, predominantly resulting from domestic accidents involving contact with stoves or hot surfaces.^1^ These contractures arise as restrictive scar formations that limit finger extension, compromise the opening of the web spaces, and can significantly impede normal hand growth and function, particularly when the palmar surface is involved.^2^^,^^3^ Globally, pediatric hand burns are a major public health issue, with restrictive scars developing in nearly 30% to 60% of children with deep palmar burns despite advances in acute care, leading to substantial long-term morbidity.^3^, ^4^, ^5^, ^6^^.^Children under five years old are especially vulnerable, and those with deep palmar thermal burns requiring surgical management are at considerable risk for developing sequelae.^7^ Early identification of risk factors and timely intervention remain pivotal to improving outcomes for this at-risk population.^8^

Beyond their frequency, palmar burns in the youngest children raise specific concerns. The hand grows rapidly during the first years of life, and a scar that appears acceptable at healing may become restrictive as the palm and the web spaces expand, so that the functional consequences of a palmar burn are often fully expressed only after several years of growth. The palm is also a small, highly specialized surface in which thumb opposition, web space opening, and digital extension must all be preserved, which makes any contracture functionally costly. Despite this, most published series rely on cross-sectional assessment at a single time point and report contracture as a simple proportion, without modeling when contractures occur or which anatomical zones drive both their development and the need for early surgery. Data resolved by palmar zone and analyzed over time therefore remain scarce, particularly in children under five years of age managed with split-thickness skin grafting. The optimal graft for the pediatric palm also remains debated, between full-thickness grafts, conventional split-thickness grafts and glabrous plantar grafts, and this controversy has so far been addressed mainly through small series with heterogeneous endpoints and short follow-up. The present work does not compare grafting techniques: it reports the long-term outcome of a cohort uniformly managed with thin split-thickness skin grafting, and its aim is to map risk within the palm rather than to rank techniques.

Our objective was to identify, in a large single-center pediatric cohort with long-term follow-up, the anatomical and clinical factors that predict post-burn palmar contracture and the factors associated with the timing of secondary corrective surgery, using time-to-event analysis to capture the temporal dynamics that cross-sectional studies cannot.

## Materials and methods

### Study design and setting

This retrospective study was conducted in accordance with the Declaration of Helsinki and with French regulations on non-interventional research. It was approved by our local ethic committee: Groupe Nantais d’Ethique dans le Domaine de la Santé (GNEDS), reference 26-125-04-24, and complied with the MR-004 reference methodology of the French Data Protection Authority (CNIL). In accordance with French law on retrospective studies, parents or legal guardians received written information and did not object to the use of their child’s data, including for the structured telephone interview used to update outcomes. No identifying information is reported.

This research employed a retrospective cohort design conducted at a single regional pediatric burn reference center. The study examined all eligible cases managed between January 1, 2006, and January 1, 2021, representing a 15-year inclusion period. The institutional setting was an interregional specialized hospital department routinely managing deep palmar burns in children. The protocol included systematic clinical follow-up to a minimum of five years post-injury, with retrospective data updated through February 2026 to ensure accurate assessment of late outcomes and sequelae. This study is reported in accordance with the Strengthening the Reporting of Observational Studies in Epidemiology (STROBE) statement for cohort studies, and a completed checklist is provided as supplementary material.^9^

### Participants

Eligible participants included all children under 5 years of age who sustained a deep palmar thermal burn from contact with a hot surface and subsequently required a split-thickness skin graft to the palmar aspect of the hand. Inclusion required confirmation of the palmar involvement, a thermal contact mechanism of injury, and identification through both diagnostic and procedural hospital coding systems.

Exclusion criteria were thermal injuries from mechanisms other than contact (such as scalds, chemical, electrical, or flame injuries), absence of palmar involvement, dorsal-only burns, incomplete medical records, and loss to follow-up or non-response at final contact. We based recruitment on a systematic review of hospital electronic records, followed by screening against the eligibility criteria described above. In cases of bilateral palmar burns, each hand was included as a separate analytic unit (Table 1).

**Table 1:** Comparison of demographic characteristics and treatment modalities among patients based on the occurrence of contractures following deep burns to the palm treated with thin-skin grafts in children under five years of age.

Population characteristics	Total N = 273	Presence of a surgical scar contracture		
		No N = 148	Yes N = 125	p-value*^3^*
**Male sex***^1^*	152 (55.7)	83 (56.1)	69 (55.2)	0.884
**Age at the time of the burn***^2^, months*	16.5 (9.7)	16.2 (6.4)	16.8 (12.6)	0.283
**Burn location***^1^*				
Entire palm (Zone 1)	177 (64.8)	95 (64.2)	82 (65.6)	0.808
Palmar surface of the fingers (Zone 2)	63 (23.1)	23 (15.5)	40 (32.0)	**0.001**
Hypothenar eminence (Zone 3)	7 (2.6)	6 (4.1)	1 (0.8)	0.130
Thumb column (Zone 4)	10 (3.7)	7 (4.7)	3 (2.4)	0.352
First web space (Zone 5)	12 (4.4)	1 (0.7)	11 (8.8)	**0.001**
Metacarpal heads (Zone 6)	37 (13.6)	31 (20.9)	6 (4.8)	**< 0.001**
**Burn etiology***^1^*				-
Barbecue	6 (2.2)	1 (0.7)	5 (4.0)	
Fireplace	2 (0.7)	2 (1.4)	0 (0.0)	
Hair straightener	6 (2.2)	3 (2.0)	3 (2.4)	
Iron	26 (9.6)	13 (8.8)	13 (10.5)	
Oven	59 (21.7)	36 (24.3)	23 (18.5)	
Fireplace insert	62 (22.8)	33 (22.3)	29 (23.4)	
Stove	93 (34.2)	50 (33.8)	43 (34.7)	
Radiator	19 (6.6)	10 (6.8)	9 (6.5)	
**Bilateral burn***^1^*	88 (32.2)	45 (30.4)	43 (34.4)	0.482
**Burn severity***^1^*				**< 0.001**
Deep second-degree	234 (85.7)	137 (92.6)	97 (77.6)	
Third-degree	39 (14.3)	11 (7.4)	28 (22.4)	
**Time between burn and STSG***^1^*				0.763
10 days or less	125 (45.8)	69 (46.6)	56 (44.8)	
More than 10 days	148 (54.2)	79 (53.4)	69 (55.2)	
**Time between burn and assessment***^2^, years*	10.7 (3.3)	10.8 (3.1)	10.6 (3.5)	0.637
**Transfer to a specialized rehabilitation center***^1^*	86 (31.5)	35 (23.6)	51 (40.8)	**0.002**
**Use of Matriderm®***^1^*	18 (6.6)	6 (4.1)	12 (9.6)	0.066
**Age***^2^***,** as of February 1, 2026, years	12.0 (3.3)	12.0 (3.2)	12.0 (3.4)	0.770
**Hand growth complete***^1^*	10 (3.7)	4 (2.7)	6 (4.8)	0.520

*^1^* n (%) ; *^2^* Mean (SD) ; *^3^* Chi-square test of independence, Wilcoxon-Mann-Whitney test, Fisher's exact test.

STSG: split-thickness skin graft.

Given the retrospective nature of data extraction and long-term clinical follow-up, the potential for observer and recall bias was minimized by cross-validating data from electronic records, paper charts, and structured telephone interviews at the time of the outcome update.

### Intervention / treatment

All included children were managed according to a standardized institutional protocol that remained unchanged over the inclusion period. Initial wound care consisted of dressing changes every 48 hours with silver sulfadiazine (Flammazine®) combined with a continuously worn static palmar extension splint, with clinical reassessment of burn depth around day 7. When spontaneous re-epithelialization was not expected, surgery was performed under general anesthesia by one of the senior plastic surgeons of the burn unit, typically between day 7 and day 14 after injury.

Tangential excision was carried out without pneumatic tourniquet use to assess the quality of the excision. A hand-held guarded knife (Goulian dermatome) was used, with the guard set to remove sequential layers of 0.2 to 0.3 mm parallel to the palmar surface. Excision was continued layer by layer until viable tissue was reached, the endpoint being a pearly white, glistening dermal bed with uniform punctate bleeding and no thrombosed vessels. Preservation of viable deep dermis was favored whenever possible, and excision was stopped at the level of the palmar aponeurosis or of the peritenon when these structures were reached, without exposure of tendons. Hemostasis was obtained with 1/1000 epinephrine-soaked gauzes and selective bipolar electrocautery, limited coagulation being used in order not to compromise the graft bed.

A sheet, non-meshed split-thickness skin graft was then harvested from the scalp with an electric dermatome set at 0.2 to 0.3 mm, after infiltration of the donor site with a tumescent solution. The graft was tailored to the defect, fenestrated when required, and secured with metallic staples. In selected cases in which excision reached the subdermal plane, a 1 mm dermal template (Matriderm® 1 mm, MedSkin Solutions Dr Suwelack AG, Billerbeck, Germany) was applied under the graft in a single-stage procedure. The hand was immobilized in a palmar extension splint and the first dressing change was performed on postoperative day 5 to 7. After complete healing, postoperative care included prescribing a compression glove to be worn 24 hours daily and scheduling physiotherapy twice weekly, focusing on scar pliability, maximal skin stretch, and prevention of contracture formation. Referral to a specialized pediatric rehabilitation center was reserved for severe or complex cases. Secondary surgical correction was indicated only for functionally significant contractures resistant to conservative treatment, in accordance with standardized institutional decision pathways. Treatment adherence was monitored through medical documentation and direct patient or parental report on follow-up contacts.

### Outcome measures

The primary outcome was the occurrence, during follow-up, of a palmar scar contracture judged to require secondary corrective surgery. A contracture was diagnosed clinically at scheduled or unscheduled visits. The indication for surgery followed a standardized institutional pathway and was retained only when a palmar scar produced a fixed limitation of active or passive joint extension, a reduction of first web space (first commissure, zone 5) opening, or a restriction of grasp that interfered with hand function and persisted despite at least six months of compression therapy and physiotherapy. The decision was made jointly by the operating surgeon and the rehabilitation team. Contractures that did not meet these criteria and were managed conservatively without functional limitation were recorded but not counted as primary events. No validated functional or range-of-motion score was applied prospectively, a point addressed in the limitations. Non-operated contractures were differentiated and not considered as such for the main endpoint because of the lack of functional impairment. The secondary outcome was the time interval from the date of skin-graft surgery to the date of first secondary corrective surgery for contracture, measured to assess timing and risk factors for earlier intervention. All outcomes were assessed within the scope of the regular clinical follow-up and updated via standardized parental or patient interview in February 2026.

### Data collection tools

Data were retrieved from multiple institutional sources. Demographics (age, sex, date of injury), burn characteristics (depth, anatomic localization, laterality, etiology), and treatment details (timing and nature of surgical procedures, use of dermal regeneration template (Matriderm® 1 mm (MedSkin Solutions Dr Suwelack AG, Billerbeck, Germany), rehabilitation pathway) were systematically extracted from digital records, operative notes, and nursing charts, using institutional coding.

For analytical purposes, the palmar surface was partitioned into six anatomical zones (Fig. 1), and burn location for each case was mapped according to detailed schematic charts completed in the original medical record. Missing or ambiguous data were resolved through manual review of paper charts and, systematically via structured telephone contact with parents or (in case of older children) the patient, focused on clarifying the presence, timing, and severity of contractures and additional interventions. Monitoring of transfers to rehabilitation specialized centers was documented prospectively by treating staff.

**Fig. 1:**
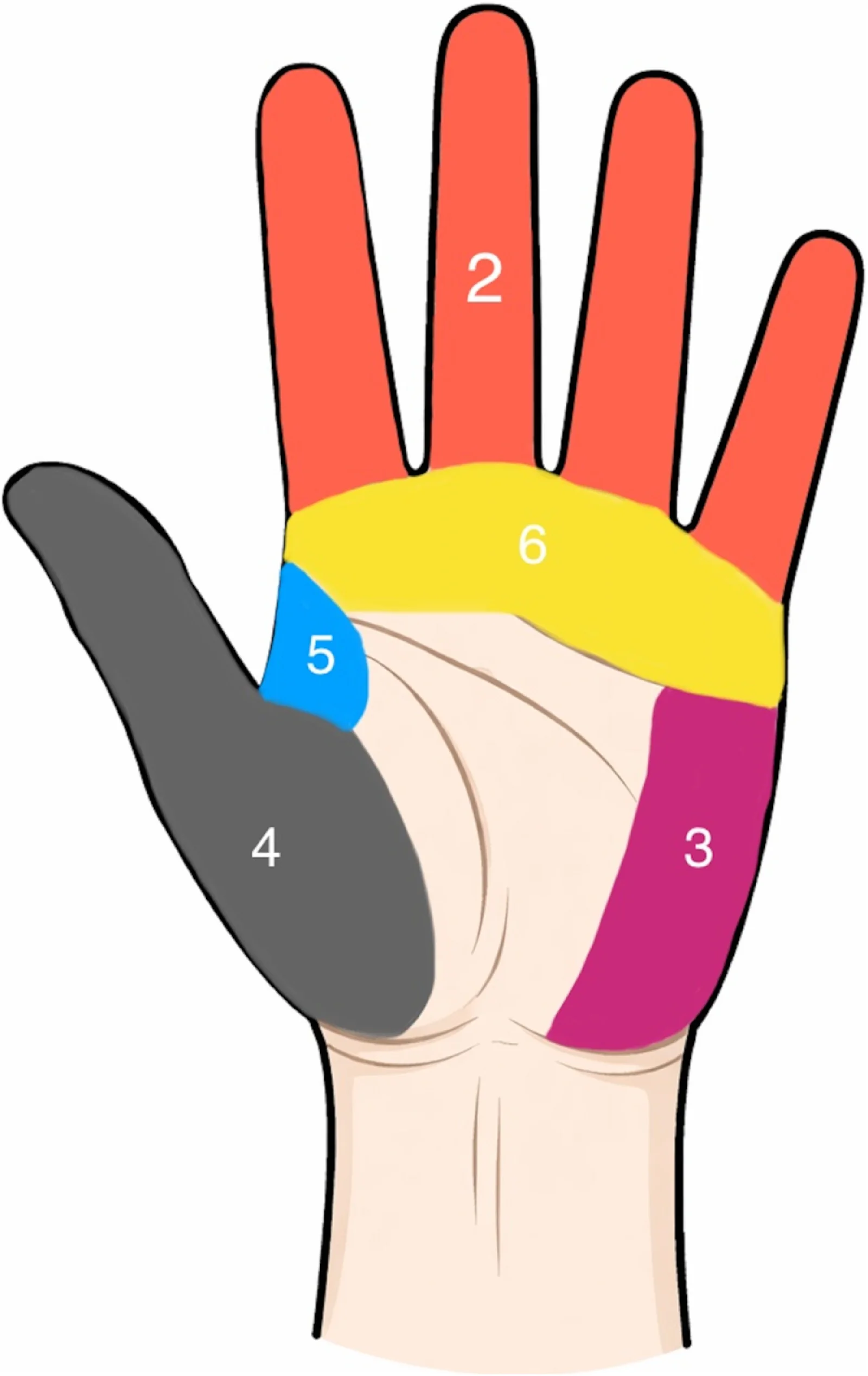
Areas of the palmar surface of the hand.

1: entire palmar surface, 2: palmar surface of the fingers, 3: hypothenar eminence, 4: thumb column, 5: first web space, 6: metacarpal heads

### Statistical analysis

All statistical analyses were conducted using R software, version 4.5.1. Data were first summarized descriptively: categorical variables as frequency counts and percentages, and continuous variables as means with standard deviations. For groupwise comparisons (e.g., patients who developed surgical contractures versus those who did not), the chi-squared or Fisher's exact test was used for qualitative variables, and the Wilcoxon-Mann-Whitney test was used for quantitative variables, based on the underlying distribution.

Multivariable risk factor analysis was performed using Cox proportional hazards survival modeling, with stepwise inclusion of variables achieving p < 0.20 in univariate screens or deemed clinically relevant. Survival curves for time to contracture were generated using the Kaplan-Meier method, with comparison between strata done using the log-rank test.

The same analytic approach was applied to the subset of patients who developed a contracture to identify predictors of earlier surgical release. Patients lost to follow-up or without contracture by the end of surveillance were censored. The threshold of significance was set at a two-sided p-value less than 0.05. Missing data were addressed by review and direct contact as above, and analyses were performed using the final available dataset.

## Results

### Participant flow

From 865 records (age <5 yo) with deep hand burn and skin graft codes (01/01/2006–01/01/2021), 232 non-contact burns and 255 dorsal-hand cases were excluded, leaving 378 palmar contact burns. After removing 62 incomplete charts and 87 non-responders, 229 children remained. Forty-four of these children had bilateral palmar burns and each contributed a second hand, so the final analytic cohort comprised 273 burned palms in 229 children. Data were updated to 02/01/2026, giving a minimum follow-up of 5 years (mean 10.7 years) for each child (Fig. 2).

**Fig. 2:**
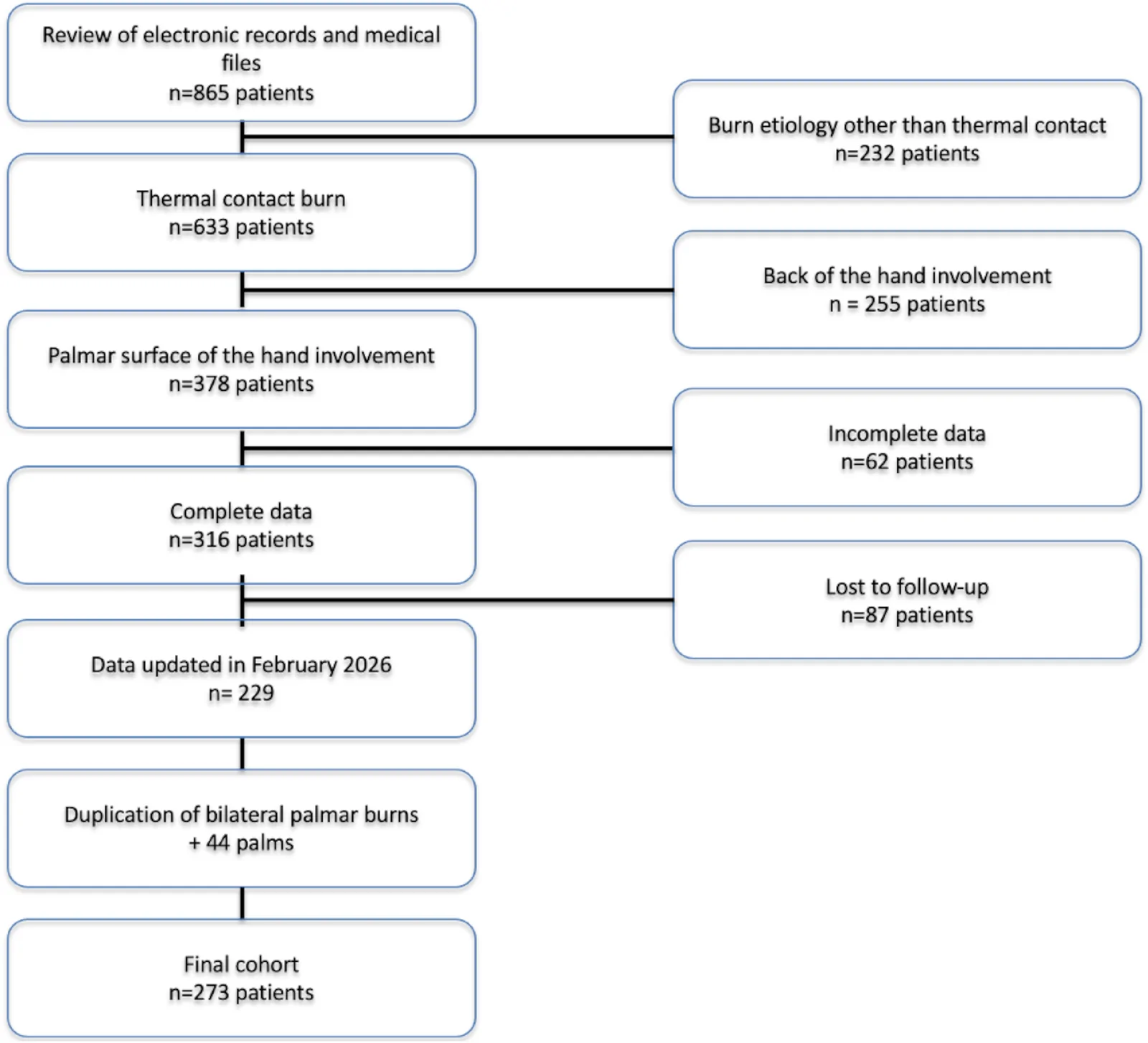
Patient selection flowchart.

Table 1 presents all baseline characteristics. During follow-up, 125 of the 273 palms (45.8%) developed a palmar scar contracture requiring secondary corrective surgery. Eighteen patients exhibited multiple contracture sites, yielding 143 distinct locations analyzed. Only 10 patients in the entire cohort (3.7%) had fully developed hands, all at age 18. Time‑to‑event analysis showed an early decline in freedom from contracture, with a median occurrence between 5 and 6 years post‑grafting; Kaplan-Meier log‑rank comparisons across evaluated factors did not demonstrate significant differences (all p > 0.05) (Fig. 3).

**Fig. 3:**
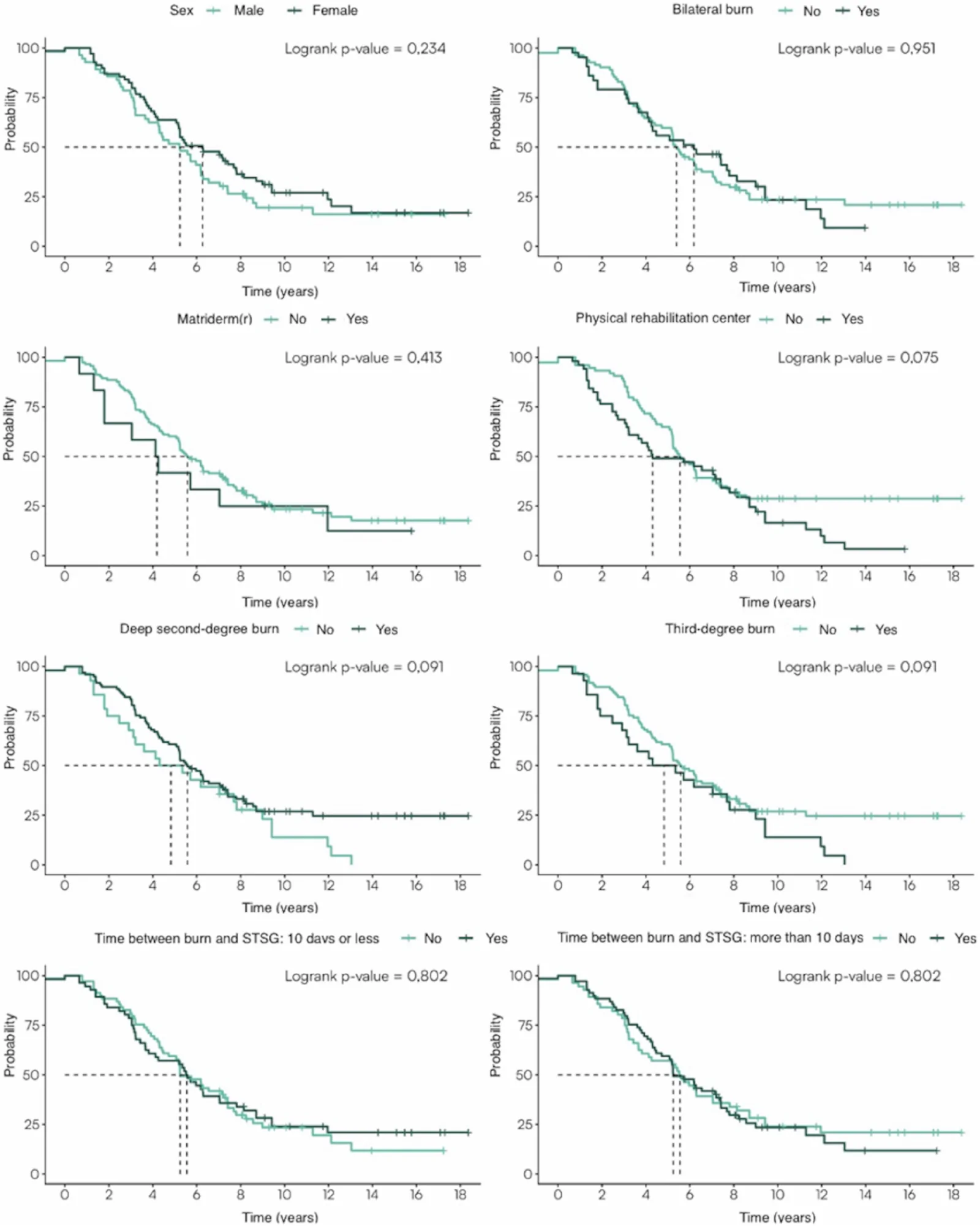
Kaplan-Meier graphs showing the probability of scar contracture formation based on various factors.

In multivariate Cox models, burns involving the first web space (Zone 5) were the strongest independent predictor for developing a palmar contracture, with an Adjusted Hazard Ratio (HRa) of 4.45 (95% CI 1.79–11.1; p = 0.001), indicating a roughly 4.5-fold increased instantaneous risk compared to other locations. Additional independent predictors included third‑degree burn severity (HRa 1.93; 95% CI 1.23–3.04; p = 0.004) and referral to a specialized rehabilitation center (HRa 1.95; 95% CI 1.26–3.02; p = 0.003), each conferring about a twofold increased risk. In contrast, involvement of the metacarpal heads (Zone 6) appeared protective, being associated with a significantly lower risk of contracture (HRa 0.32; 95% CI 0.11–0.92; p = 0.034), or a 68% reduction in instantaneous risk compared to reference. Notably, some variables that were significant in univariate analysis lost significance in the multivariate context; for example, involvement of the palmar digits (Zone 2) had an adjusted HRa of 1.56 (95% CI 0.89–2.73; p = 0.122). Similarly, use of Matriderm®, though initially associated with risk in univariate analysis (HRa 1.95; 95% CI 1.07–3.53; p = 0.029), was not significant after adjustment (HRa 0.97; 95% CI 0.51–1.85; p = 0.983). No significant associations were observed for time from burn to graft >10 days (HRa 1.12; 95% CI 0.79–1.60; p = 0.505), sex (HRa 0.96; 95% CI 0.67–1.36; p = 0.832), bilaterality (HRa 1.20; 95% CI 0.83–1.74; p = 0.321), or age at burn (HRa 1.00; 95% CI 0.98–1.01; p = 0.606). These multivariate findings emphasize anatomical location and burn depth as the principal independent predictors of contracture. Referral to a specialized rehabilitation center remained statistically associated with contracture but is interpreted below as a marker of initial severity rather than a causal factor (Table 2).

**Table 2:** Univariate and multivariate analysis using the Cox model of predictors of scar contracture.

Population characteristics	Univariate analysis		Multivariate analysis	
	HR (CI95%)	p-value	HRa (CI95%)	p-value
**Sex**	0.96 [0.67 - 1.36]	0.832	-	
**Time between burn and STSG***^1^*				
10 days or less	*Reference*		-	
More than 10 days	1.12 [0.79 - 1.60]	0.505	-	
**Burn location***^1^*				
Entire palm (Zone 1)	0.96 [0.66 - 1.39]	0.812	0.67 [0.34 - 1.35]	0.343
Palmar surface of the fingers (Zone 2)	2.08 [1.42 - 3.04]	**< 0.001**	1.56 [0.89 - 2.73]	0.122
Hypothenar eminence (Zone 3)	0.26 [0.04 - 1.86]	0.213	0.14 [0.02 - 1.18]	0.071
Thumb column (Zone 4)	0.65 [0.21 - 2.05]	0.502	0.72 [0.21 - 2.45]	0.604
First web space (Zone 5)	4.62 [2.46 - 8.67]	**< 0.001**	4.45 [1.79 - 11.1]	**0.001**
Metacarpal heads (Zone 6)	0.27 [0.12 - 0.61]	**0.002**	0.32 [0.11 - 0.92]	**0.034**
**Bilateral burn***^1^*	1.20 [0.83 - 1.74]	0.321	-	
**Burn severity***^1^*				
Deep second-degree	*Reference*		*Reference*	
Third-degree	2.45 [1.60 - 3.75]	**< 0.001**	1.93 [1.23 - 3.04]	**0.004**
**Transfer to a specialized rehabilitation center***^1^*	1.97 [1.37 - 2.83]	**< 0.001**	1.95 [1.26 - 3.02]	**0.003**
**Use of Matriderm®***^1^*	1.95 [1.07 - 3.53]	**0.029**	0.97 [0.51 - 1.85]	0.983
**Age at the time of the burn***^2^, months*	1.00 [0.98 - 1.01]	0.606	-	

HR = hazard ratio, HRa = adjusted hazard ratio, CI = confidence interval.

### Time to corrective surgery

Among patients who underwent secondary corrective surgery for palmar contracture (n = 125), the interval from skin graft to first scar contracture correction averaged 3.2 years (SD 2.9). In Cox models of time to surgery, burns located in the hypothenar eminence (Zone 3) and the first web space (Zone 5) emerged as the strongest independent predictors of earlier intervention. Specifically, hypothenar involvement (Zone 3) was associated with the highest adjusted instantaneous risk (HRa 18.5; 95% CI 2.09–164; p = 0.009), while first web space involvement also demonstrated a significantly higher hazard (HRa 2.84; 95% CI 1.06–7.60; p = 0.038) for earlier surgical management. Palmar digits (Zone 2) showed a significant association on univariate analysis (HRa 1.67; 95% CI 1.10–2.55; p = 0.016), but this association lost significance after adjustment (HRa 1.75; 95% CI 0.94–3.24; p = 0.077). Total palmar involvement (Zone 1) showed a nonsignificant trend toward lower hazard (univariate HRa 0.67; 95% CI 0.44–1.02; p = 0.062; adjusted HRa 0.91; 95% CI 0.42–1.96; p = 0.812), while thumb column (Zone 4) and metacarpal heads (Zone 6) were not independently associated with timing (Zone 4 HRa 2.65; 95% CI 0.75–9.40; p = 0.131; Zone 6 HRa 0.75; 95% CI 0.19–2.92; p = 0.703). Other tested factors, including sex, age at injury (per year increase: HRa 0.99; 95% CI 0.98–1.01; p = 0.412), bilateral burns, third-degree depth, time from burn to graft >10 days, transfer to specialized rehabilitation, and use of Matriderm®, did not reach statistical significance for earlier secondary intervention (Table 3).

**Table 3:** Univariate and multivariate Cox proportional hazards analysis of factors associated with the time to secondary surgical treatment of scar contracture (n = 125).

Population characteristics	Univariate analysis		Multivariate analysis	
	HR (CI95%)	p-value	HRa (CI95%)	p-value
**Sex**	0.78 [0.52 - 1.17]	0.212	-	
**Time between burn and STSG***^1^*				
10 days or less	*Reference*		-	
More than 10 days	1.05 [0.70 - 1.58]	0.803	-	
**Burn location***^1^*				
Entire palm (Zone 1)	0.67 [0.44 - 1.02]	0.062	0.91 [0.42 - 1.96]	0.812
Palmar surface of the fingers (Zone 2)	1.67 [1.10 - 2.55]	**0.016**	1.75 [0.94 - 3.24]	0.077
Hypothenar eminence (Zone 3)	10.8 [1.39 - 83.4]	**0.023**	18.5 [2.09 - 164]	**0.009**
Thumb column (Zone 4)	2.86 [0.89 - 9.20]	0.078	2.65 [0.75 - 9.40]	0.131
First web space (Zone 5)	1.98 [1.02 - 3.84]	**0.044**	2.84 [1.06 - 7.60]	**0.038**
Metacarpal heads (Zone 6)	0.51 [0.16 - 1.62]	0.301	0.75 [0.19 - 2.92]	0.703
**Bilateral burn***^1^*	1.01 [0.67 - 1.54]	0.982	-	
**Burn severity***^1^*				
Deep second-degree	*Reference*		*Reference*	
Third-degree	1.47 [0.94 - 2.31]	0.092	1.29 [0.77 - 2.15]	0.302
**Transfer to a specialized rehabilitation center***^1^*	1.44 [0.96 - 2.16]	0.075	1.49 [0.90 - 2.45]	0.124
**Use of Matriderm®***^1^*	1.31 [0.68 - 2.53]	0.403	-	
**Age at the time of the burn***^2^, months*	0.99 [0.98 - 1.01]	0.412	-	

HR = hazard ratio, HRa = adjusted hazard ratio, CI = confidence interval.

## Discussion

To our knowledge, this is among the largest pediatric series of deep palmar contact burns analyzed with time-to-event methods. Its contributions are threefold: a zone-resolved risk map of the palm, including a previously undescribed protective association for metacarpal head involvement; an explicit analysis of the timing of secondary surgery, identifying hypothenar and first web space burns as drivers of early intervention; and a dedicated analysis of bilateral involvement over a long follow-up.

The findings of this study align with prior reports indicating a high prevalence of post-burn contracture in children with deep palmar burns, with published rates typically ranging from 30% to 60%, similar to the observed rate in this cohort. The identification of third-degree burn depth and first commissural involvement as major independent risk factors for contracture mirrors previous literature, which emphasizes anatomical location and burn depth over other variables.^10^, ^11^, ^12^, ^13^, ^14^ The strong association between first web space involvement and contracture risk, as well as a functional imperative for early secondary surgery in this zone, also parallels the established understanding that the first web space is particularly prone to sequelae due to its critical role in thumb opposition and prehension.^10^^,^^14^^,^^15^ Conversely, the protective effect observed for burns localized to the metacarpal heads is not clearly described in existing studies, representing a point of divergence and potentially novel insight. Additionally, the study's finding that surgical timing after burn injury and the use of the dermal substitute Matriderm® do not independently influence contracture risk is consistent with the literature's lack of robust evidence for these factors.^16^, ^17^, ^18^ Finally, the use of multivariate time-to-event analysis addresses a recognized gap, as most previous studies rely on cross-sectional methods and do not assess temporal dynamics.^19^, ^20^, ^21^

The association between referral to a specialized pediatric burn rehabilitation center and the development of contractures (HRa=1.95) should be interpreted with caution. It remains an indirect marker of initial severity rather than a causal factor, reflecting biases in referral criteria and disease severity. Indeed, patients referred to specialized centers had more extensive and deeper injuries, requiring intensive rehabilitation and/or a social context that was not conducive to adherence to the care protocol necessary to limit the occurrence of sequelae. This phenomenon is well described in observational studies in burn surgery.^18^^,^^20^^,^^21^ and underscores the importance of distinguishing prognostic factors from organizational factors.

Our cohort was uniformly managed with thin split-thickness skin grafts harvested from the scalp, and the 45.8% rate of contracture requiring secondary surgery observed over a mean follow-up of 10.7 years must be read in the light of the ongoing debate on graft selection in the pediatric palm. Comparative data remain limited and partly contradictory. In the only prospective independent evaluation, Chandrasegaram et al.^6^ found no functional difference between full-thickness and split-thickness grafts, and Pensler et al.^22^ had previously reported comparable function and a comparable number of procedures with both techniques. Conversely, the systematic review and meta-analysis by Liu et al.^1^ concluded that full-thickness grafts are associated with a lower occurrence of post-graft contracture and with a lower need for surgical release, while split-thickness grafts performed better on scar, color and aesthetic assessment.

The two series highlighted during review deserve specific comment. Baur et al.^23^ reported that glabrous split-thickness grafts taken from the instep were prone to motion-limiting contracture in growing children and concluded that full-thickness grafts should remain the reference standard, although only three children in that series received a glabrous split-thickness graft. Elrod et al.^24^ compared retro auricular full-thickness grafts with thick plantar glabrous split-thickness grafts and found the glabrous grafts superior for pigmentation, vascularity and observer-rated scar quality, whereas full-thickness grafts were more pliable. Taken together, these data suggest that the relevant distinction may be less split-thickness versus full-thickness than glabrous versus non-glabrous, and thin versus thick dermal content, the dermal component being the main determinant of secondary contraction.^25^, ^26^, ^27^ The grafts used in our cohort were thin and non-glabrous, which plausibly contributed to the contracture rate we report and may partly explain why it lies in the upper part of published estimates.

Two features of our design temper any direct comparison with these series. First, our endpoint was deliberately restrictive: only contractures producing a fixed functional limitation and leading to secondary surgery were counted, whereas several published series report any clinically detectable contracture or any revision procedure. Second, our follow-up was substantially longer than in most comparative studies, and contractures in this age group continue to appear with growth, so that shorter series are expected to underestimate the cumulative rate. Our figure is therefore conservative in definition but higher in magnitude than most cross-sectional reports, and the two effects should not be confused.

We could not perform an internal comparison between graft types, since a single standardized protocol was applied throughout the inclusion period. This uniformity is a methodological strength for the identification of anatomical risk factors, because graft type cannot confound the zone-resolved estimates we report, but it is also a limitation, and the present data cannot be used to rank grafting techniques. In our view, the zone-resolved risk map reported here is complementary to the graft selection debate: whichever graft is chosen, first web space involvement and third-degree depth identify the hands that require the closest surveillance and the earliest reconstructive planning.

The decision to set the evaluation threshold at 10 days is debatable. It is consistent with our center's practice, which involves changing dressings every 2 days with silver sulfadiazine (Flammazine®) in combination with a 24-hour palmar extension splint, re-evaluation around 7 days after the burn, and performing a split-thickness skin graft in the second week following the burn. We chose this 10-day cutoff based on the literature, which remains inconsistent, and our previous work on the face ,^28^ which is an area of high functional importance, similar to the palm .

Furthermore, in our cohort, only two patients underwent excision and grafting within 72 hours (0.73%), 12 between 3 and 7 days (4.40%), 204 between 7 and 14 days (74.72%), and 55 > 14 days (20.15%). The mean and median times to excision and split-thickness skin grafting were 10.2 and 10.4 days, respectively, further supporting the selection of a 10-day cutoff to define our evaluation groups.

No study has specifically analyzed the impact of bilateral burns to a child’s palms on the risk of developing contracture-like sequelae. A few studies described bilateral palm involvement in their cohorts.^15^^,^^24^^,^^25^^,^^29^ but in smaller proportions than in our study (88 palms, or 44 patients). In this cohort, bilateral involvement was not associated with a higher risk of secondary surgery for contracture.

Unlike cross-sectional analyses, this approach allows for the incorporation of the temporal dimension of bridle-type complications. Kaplan-Meier curves show an early onset of adhesions during follow-up (median at 5–6 years), with an initial rapid decline followed by a secondary plateau, indicating a stabilization of risk. Unadjusted Kaplan-Meier comparisons were not statistically significant, whereas multivariable Cox modeling identified independent associations after adjustment for confounding. This discrepancy is explained by the fact that the Kaplan-Meier curves are based on an unadjusted analysis that does not account for confounding factors. The Cox model, by simultaneously adjusting for multiple variables, allows for the identification of independent effects. This methodological point is essential and strengthens the internal validity of the results.

This retrospective single-center design introduces limitations. The monocentric approach may limit generalizability due to specific institutional protocols and patient populations. Data collection relied on medical chart review and telephone follow-up, potentially contributing to information bias, incomplete or inconsistent records, and reliance on subjective self- or parent-report for some outcomes. Due to the low proportion of patients whose hands have stopped growing (3.7%), most patients were not followed to the end of hand growth, risking underestimation of late sequelae. This will require us to continue our work over the next few years, specifically by tracking this population to eliminate this bias. A lack of standardized, objective, functional outcome assessment may further bias results. Since our burn center is the only one in the western quarter of the country, many patients could not be recalled for a follow-up visit to undergo an additional clinical examination. These issues should be considered when interpreting the findings.

Each hand was treated as an independent unit, whereas the 44 children with bilateral burns each contributed two correlated hands. We did not use a cluster-robust or mixed-effects model to account for this within-child correlation, which may have slightly underestimated the width of the confidence intervals. Given the limited size of the bilateral subgroup relative to the full cohort, any impact on the reported estimates is likely minor.

Finally, the exclusive use of thin non-glabrous split-thickness grafts precludes any internal comparison with full-thickness or glabrous grafting, and our contracture rate should not be extrapolated to units using those techniques.

The generalizability of these results is influenced by the study's monocentric, retrospective design and the specific national regional referral context. While the cohort is relatively large and follows standardized protocols with long-term follow-up, the findings primarily reflect clinical practice and patient characteristics in a single specialized center. Inclusion was limited to children under five with deep contact palmar burns, which enhances internal validity but may restrict applicability to older children, other injury mechanisms, or less specialized settings. Our findings have significant implications for the management of deep palmar thermal burns in children. By identifying the first web space involvement and third-degree burn depth as independent predictors of post-burn contracture; the hypothenar region and the first web space as areas at risk for early secondary surgery, our study enables more precise risk stratification at admission. Early identification of high-risk cases enables intensified surveillance and more tailored rehabilitation protocols, particularly in anatomically vulnerable zones.

## Conclusion

Deep palmar burns in children remain associated with a high risk of functional sequelae despite specialized care. The first web space and third degree depth are the major determinants of contracture development. Survival analysis highlights the early onset of these complications, underscoring the need for prolonged, targeted follow-up. These findings apply to palms resurfaced with thin split-thickness skin grafts, and comparative studies against full-thickness and glabrous grafts, using standardized functional endpoints and follow-up to skeletal maturity, remain necessary. These results support risk stratification from the initial phase and a multidisciplinary approach tailored to the child's growth, with a personalized follow-up tailored to the burn and patient characteristics.

## Funding

This research did not receive any specific grant from funding agencies in the public, commercial, or not-for-profit sectors.

## Ethical approval

This study was approved by the local ethics committee, Groupe Nantais d'Éthique dans le Domaine de la Santé (GNEDS), under reference 26-125-04-24. It was conducted in accordance with the Declaration of Helsinki and complied with the MR-004 reference methodology of the French Data Protection Authority (CNIL). In accordance with French legislation on retrospective non-interventional research, parents or legal guardians received written information and did not object to the use of their child's data, including for the structured telephone interview used to update outcomes. No identifying information is reported.

## Clinical trial number

Not applicable.

## Use of generative AI

Not applicable.

## Informed consent

In accordance with French law on retrospective non-interventional research, parents or legal guardians received written information and did not object to the use of their child’s data. No separate written informed consent was required.

## CRediT author statement

UL: conceptualization, methodology, formal analysis, writing original draft. FT: formal analysis, writing original draft. MC: supervision, writing review and editing. EE: supervision, writing review and editing. PP: supervision, writing review and editing. All authors approved the final manuscript.

## Declaration of competing interest

The authors declare that they have no known competing financial interests or personal relationships that could have appeared to influence the work reported in this paper.

## Data Availability

The data that support the findings of this study are available from the corresponding author upon reasonable request. They are not publicly available because they contain information that could compromise the privacy of pediatric patients.
